# Integrated multi-omics profiling reveals phenotype- and tissue-specific host-microbiota interactions in paired tumor and peritumoral tissues of advanced gastric cancer patients from Northwest China

**DOI:** 10.3389/fcimb.2026.1763765

**Published:** 2026-04-20

**Authors:** Anqi Wang, Qi Wang, Ting Zhang, Guoqing Qi, Wen Ren, Wenji Tian, Juanjuan Chen

**Affiliations:** 1 NHC Key Laboratory of Diagnosis and Therapy of Gastrointestinal Tumor, Gansu Provincial Hospital, Lanzhou, Gansu, China; 2 Cuiying Biomedical Research Center, The Second Hospital & Clinical Medical School, Lanzhou University, Lanzhou, Gansu, China; 3 Department of Anesthesiology and Surgery, The Second Hospital & Clinical Medical School, Lanzhou University, Lanzhou, Gansu, China; 4 The Second Clinical Medical College, Lanzhou University, Lanzhou, Gansu, China; 5 Department of Gastroenterology, The Second Hospital & Clinical Medical School, Lanzhou University, Lanzhou, Gansu, China; 6 Department of Oncology, Gansu Provincial Central Hospital, Lanzhou, Gansu, China

**Keywords:** advanced gastric cancer, differentially expressed genes, *Helicobacter pylori*, host-microbe interactions, Lauren classification, Northwest Chinese, transcriptomic and metagenomic analyses

## Abstract

**Background:**

Advanced gastric cancer (AGC) exhibits a high incidence in Northwest China, largely attributed to region-specific dietary patterns and environmental exposures. Its pathogenesis involves complex host-microbiota crosstalk, which has not yet been comprehensively elucidated through integrated multi-omics approaches. Herein, we employed trasncriptomic and shotgun metagenomic sequencing on paired tumoral and peritumoal mucosal tissues from 88 AGC patients in Northwest China. Our aim was to systematically characterize host gene expression profiles, the composition and functional potential of the gastric mucosal microbiota, and their intricate interrelationships.

**Results:**

Transcriptomic profiling clearly distinguished tumoral from peritumoral regions (PERMANOVA, R^2^ = 0.24, P = 0.0001), with 8870 differentially expressed genes (DEGs) identified between the two tissue types. Tumor tissues harbored 8377 up-regulated DEG, which were enriched in extracellular matrix (ECM) organization, cell cycle regulation, signaling transduction, and inflammatory pathways (e.g., PI3K-Akt, IL-17 signaling). In contrast, peritumoral tissues showed 493 up-regulated DEGs primarily associated with metabolic processes. Host gene expression was significantly modulated by Lauren classification in tumoral mucosa (P = 0.025) and by *Helicobacter pylori* (Hp) infection in peritumoral tissues (P = 0.0424). Hp-infected tissues exhibited 65 up-regulated DEGs linked to transcriptional misregulation in cancer, inflammation, immune activation and mitochondrial pathways. Lauren subtypes displayed distinct transcriptomic signatures: intestinal-type AGC was enriched in metabolic processes, diffuse-type in immune and signal transduction pathways, and mixed-type in Ras/MAPK/ErbB and NF-κB signaling pathways. Correlation analysis between the 8870 DEGs and seven differentially abundant bacterial species (e.g., *Serratia surfactantfaciens*, *Pseudomonas protegens*, *Prevotella jejuni*, and *Streptococcus infantis*) revealed 13199 significant correlations. Among these, *S. surfactantfaciens* and *P. protegens* exhibited the strongest connectivity with host genes. Functionally, the correlated DEGs were involved in ECM structure, cell cycle progression, immune and inflammatory responses, cellular proliferation and differentiation, and metabolic processes.

**Conclusions:**

Our findings demonstrated phenotype- and tissue-specific regulation of host gene expression in AGC and revealed extensive host-microbe interactions. This work fills a critical gap in multi-omics research on AGC in the Northwest Chinese population and suggests potential diagnostic and therapeutic targets for AGC.

## 1 Introduction

Advanced gastric cancer (AGC) is a major cause of global cancer-related deaths (Ratti et al., 2024; Sundar et al., 2025). Although therapeutic advances, the five-year survival rate remains low at 30%-40%, highlighting the need to elucidate the molecular mechanisms driving gastric cancer (GC) progression (Sundar et al., 2025). Both dysregulated gene expression and gastrointestinal microbiota dysbiosis are crucial in GC development, influencing tumor initiation, progression, and treatment response (Dai et al., 2021; Park et al., 2022; Nobels et al., 2025; Philip et al., 2025; Qian et al., 2025).

Gene expression profiles, involving oncogenes, tumor suppressor, and signaling pathways, drive key cancer hallmarks such as uncontrolled proliferation, invasion, metastasis, and apoptosis resistance (Loe et al., 2023; Jiang et al., 2024; Zhu et al., 2025). Aberrant activation of key pathways such as PI3K/Akt, MAPK, and Wnt/β-catenin promotes malignant features and has been linked to poor therapeutic outcomes (Chiurillo, 2015; Fattahi et al., 2020; Lei ZN. et al., 2022; Han et al., 2024; Yuan et al., 2024). Transcriptomic studies have identified gene signatures associated with GC subtypes and outcomes (Totoki et al., 2023; Balikci and Kucukakcali, 2025), yet the regulatory networks within the tumor microenvironment (TME) of AGC are incompletely characterized.

The gastric microbiota, though historically understudied due to low abundance caused by the harsh acidic environment, is now recognized as a key component of the TME (Dai et al., 2021; Zhou et al., 2023; Liu Z. et al., 2024). However, most of current studies focus on the gut microbiota-GC axis (Elghannam et al., 2025; Huo et al., 2025; Nobels et al., 2025). Beyond *Helicobacter pylori* (*H. pylori*, Hp), Taxa such as *Streptococcus*, *Lactobacillus*, and *Prevotella*, along with microbial metabolites including short-chain fatty acids and lipopolysaccharides (Dai et al., 2021; Lei C. et al., 2022), can influence host signaling, immunity, and gene expression to aggravate GC progression (Ferreira et al., 2018; Hu et al., 2018; Yang et al., 2023; Santacroce et al., 2024).

Emerging evidence indicates bidirectional crosstalk between the microbes and host gene expression as a critical axis in GC pathogenesis (Qian et al., 2025). Microbes can regulate host gene activity through epigenetic mechanisms (e.g., DNA methylation, histone acetylation) or transcription factors (e.g., NF-κB, STAT3) (Wang et al., 2022; Lei et al., 2025), while host genetic variations in pattern recognition receptors (e.g., TLRs) or immune regulators can reshape the mucosal landscape and microbial composition (Woo and Alenghat, 2017; Chen et al., 2025). This interaction may form a feed-forward loop that accelerates GC progression and therapy resistance, a dynamic still poorly understood in AGC.

Despite these insights, most existing research on GC have examined transcriptomic signatures and microbial compositions separately, with limited research integrating both aspects in AGC. Moreover, Northwest China is a region with a high incidence of GC, with most patients are diagnosed at an advanced stage. The population in Gansu and other Northwest Chinese regions has a typical dietary pattern of high salt, pickled foods, and low fresh fruit/vegetable intake and enviromental exposures of relatively dry climate, high air pollution, and low selenium content in soil and food. High salt and pickled foods were reported to contribute the gastric cancer by increasing gastric pH, altering the gastric microbial composition, and host gene mutation (Fang et al., 2015; Yoo et al., 2020; Wu et al., 2021; Liu et al., 2025). Dry climate, high altitude, and low selenium can also affect the gastric microbiota and host immune function (Liu D. et al., 2024; Ma et al., 2025; Qi et al., 2025). These factors underscore an urgent need for improved diagnosis, treatment and early prevention strategies for GC in this region. This study aims to characterize the gene expression profiles of AGC patients and investigate their associations with gastric microbiota. By integrating transcriptomic and metagenomic data, we seek to reveal key interaction networks between host gene expression and microbial communities, and to explore their potential roles in tumor progression and clinical outcomes. Our work will provide novel mechanistic insights, identify combined biomarkers for early diagnosis or prognosis, and lay a foundation for personalized therapeutic strategies in AGC.

## 2 Methods

### 2.1 The aim, design and setting of the study

**Figure 1:**
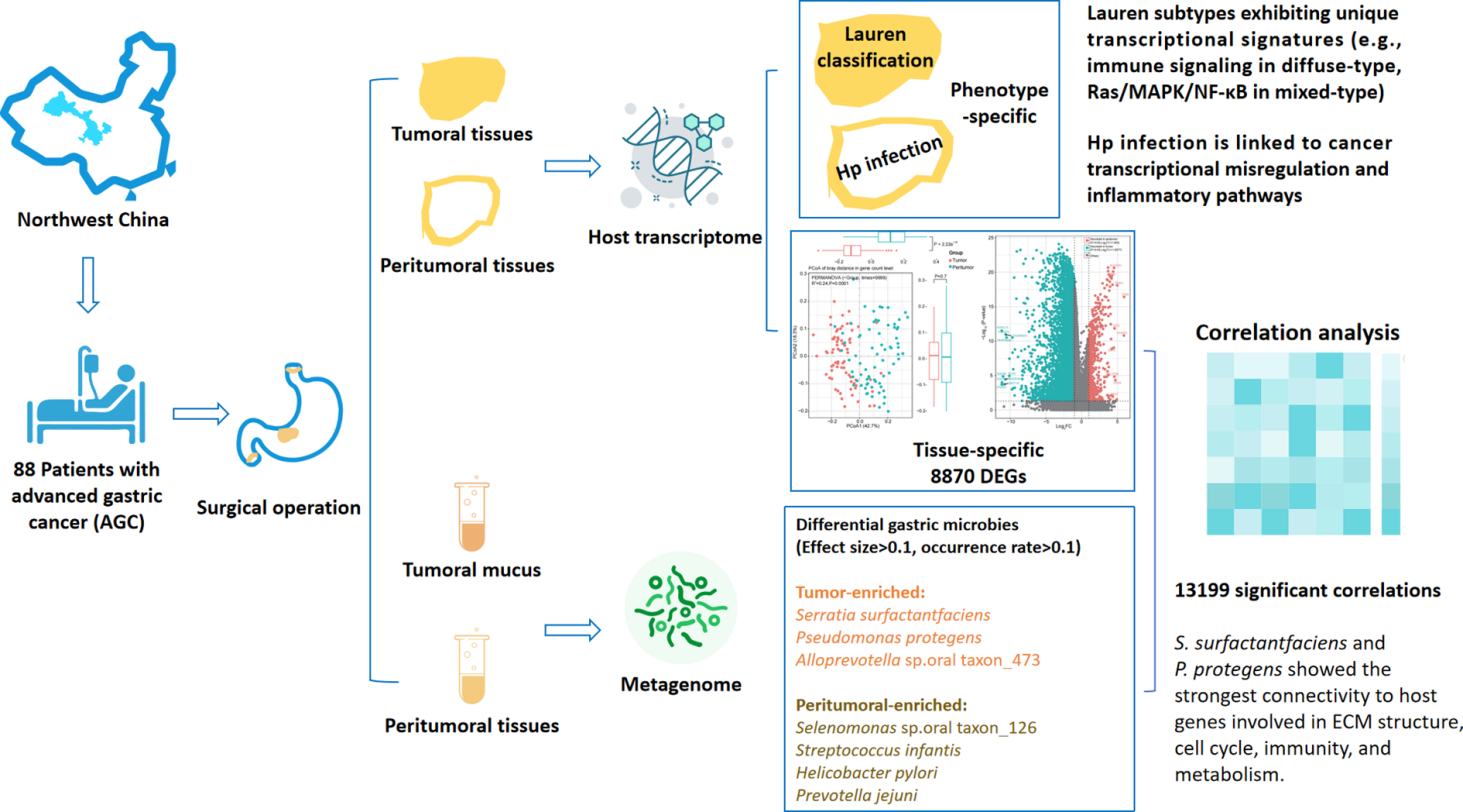
Graphical abstract of the present study.

This cross-sectional study study aims to characterize the expression profiles of gastric-related genes and the compositional features of the gastric mucosal microbiota, as well as their intricate crosstalk in AGC patients residing in Northwest China (**Figure 1**). Tumoral and peritumoral mucosal tissues, along with mucus samples, were collected from all participants to analyze the gastric gene expression patterns and characterize the gastric microbial communities (**Supplementary Table 1**).

### 2.2 Sample size justification

To enhance the reliability of the research results, G*Power 3.1 and MicrobiomeAnalyst were used to calculate the exact sample size. Following formula was used with parameters d=0.8, r=0.6, 1-β=0.90 (90% potency) and 20% drop rate.


$$\text{n}=\;\frac{2(1-r)\sigma^{2}{\left(Z_{\alpha /2}+Z_{\beta }\right)}^{2}}{d^{2}}\times (1+\text{drop rate)}$$

σ represents the standard deviation of α diversity, taking the general value of gastric cancer research from 1.0 to 1.2; Z_α/2_ = 1.96, Z_β_=0.84 (80% potency) and 1.28 (90% potency).

For transcriptomic analysis (two-independent sample t-test for α-diversity and DEG detection): With α=0.05, effect size d=0.8 (large effect, based on pre-experimental and published gastric cancer transcriptomic data), and power (1-β)=0.90, the required minimum sample size is 34 per group;

For metagenomic and correlation analysis: Based on MicrobiomeAnalyst’s power analysis for microbial differential abundance detection, with a sample size of 60 (tumor) and 53 (peritumoral), the power for detecting differentially abundant bacterial species (effect size ≥0.5) is 0.89, and the power for Spearman’s correlation analysis between microbes and host genes is 0.92 (based on the number of DEGs and microbial species in this study).

### 2.3 Subjects and clinical phenotypes information

A total of 88 AGC patients were recruited from Lanzhou University Second Hospital, with female:male=12:76; mean age: 59.01 ± 9.23 years; mean BMI: 22.65 ± 2.86. Comprehensive clinical phenotypes including sex, age, weight, height, body mass index (BMI), cancerous site, surgical method, tumor staging, lauren types (diffuse-, intestinal and mixed types), differentiation degree, Hp infection, ECOG/CCI score), treatment history (Whether to undergo chemotherapy or not), and comorbidities were collected and analyzed (**Supplementary Table 1A**).

### 2.4 Sample collection and storage

Paired tumoral and peritumoral mucosal samples (≥5 cm from the tumor margin) were collected into 1.5 mL sterile tubes during surgery, immediately flash-frozen in liquid nitrogen, and stored at -80°C. Mucus samples were scraped into 2 ml sterile tubes using a sterile spoon and promptly stored at -80°C.

### 2.5 Transcriptomic sequencing and analysis

Transcriptomic sequencing was performed on 73 tumoral and 73 peritumoral samples, 58 of these were paired (**Supplementary Table 1B**), following the procedures described previously (Li et al., 2024), including RNA extraction, library preparation, high-throughput sequencing, and bioinformatic analysis.

#### 2.5.1 Total RNA extraction and quality control

Total RNA was extracted from the frozen gastric cancer tissues using Trizol reagent (Thermo Fisher Scientific, Carlsbad, CA, USA) and following the manufacturer’s instructions, and DNase I was used to digest residual genomic DNA to eliminate interference. The concentration and purity of the extracted total RNA were detected by NanoDrop ND2000 spectrophotometer (Thermo, USA), with the criteria of OD260/OD280 between 1.8 and 2.0 and OD260/OD230 ≥2.0. The integrity of RNA was verified by 1% agarose gel electrophoresis, and only RNA samples with clear 28S and 18S bands (28S/18S ≥1.8) and RNA integrity number(RIN) ≥7.0 were used for subsequent experiments.

#### 2.5.2 Library construction

mRNA was enriched from the qualified total RNA using magnetic beads with Oligo(dT) to remove rRNA and other non-coding RNA. The enriched mRNA was used for transcriptomic library construction using the TruSeq RNA Library Prep Kit v2 (Illumina, San Diego, CA, USA). The qualified libraries were sequenced on the NovaSeq 6000 Illumina platform to generate 125 bp/150 bp paired-end reads.

#### 2.5.3 Quality control and reads mapping

FastQC was used to filter low-quality reads (reads with N content > 5%, low-quality base ratio > 20%). Adapter-polluted reads and duplicate reads were filtered out by Trim Galore software to obtain high-quality clean reads. HISAT2 v2.2.1 was used to map the filtered reads to the human reference genome.

#### 2.5.4 Differential expression analysis

The counts value of each gene was calculated using the HTSeq v0.9.1 package, followed by normalization to TPM (Transcripts Per Kilobase of exon model per Million mapped reads) values. Differentially expressed genes (DEGs) were screened using DESeq2 v1.38.3 software with the criteria of P < 0.05 and |log_2_FC| >1. Benjamini-Hochberg (BH) method to adjust the P-value and marked as Q-value in **Supplementary Table 2A**. Ggvolcano package in R 4.0.3.6 was used to perform a bidirectional clustering analysis of all the different genes in the samples.

#### 2.5.5 Permutational Multivariate Analysis of Variance (PERMANOVA) of the effect of clinical phenotypes on gene expression

PERMAONOVA was performed to evaluate the effect of various clinical phenotypes on gastric gene expression in tumoral and peritumoral tissues, respectively. Lauren classification and Hp infection were found to be significantly affect the gastric gene expression in tumoral and peritumoral tissues, respectively. For Lauren type, 32 diffuse-type, 27 intestinal-type and 20 mixed-type were included for differential analysis. For Hp infection, 31 Hp-negative and 57 Hp-positive samples were included for differential analysis.

#### 2.5.6 Functional enrichment and pathway analysis

GO annotation, KEGG enrichment analysis, and Gene Set Enrichment Analysis (GSEA) and Gene Set Variation Analysis (GSVA) were performed to explore the function of the differentially expressed genes using clusterProfiler R package in R v. P-value < 0.05 was considered significant enrichment), and the GO terms associated with significant DEGs were used to determine the main functions of the DEGs. For the KEGG pathway analysis, CLUSTERPROFILER v3.4.4 was used to conduct KEGG pathway enrichment analysis of the DEGs, and significantly enriched pathways with P < 0.05 were determined. Ggplot2 and pheatmap package in R v4.0.3.6 was used to visualize the results.

### 2.6 Shotgun metagenomic sequencing and analysis

Shotgun metagenomic sequencing was carried out in accordance with an established protocol (Wang et al., 2025), involving microbial DNA extraction, paired-end library construction, and DNBSEQ-T7-based high-throughput sequencing.

#### 2.6.1 DNA extraction and quality control

Gastric mucus samples were thawed on ice for DNA extraction using the FastDNA™ Spin Kit for Soil extraction kit (MP Biomedicals) in accordance with the manufacturer’s instructions.During the extraction process, RNase A was added to remove residual RNA, and protease K was used to decompose protein impurities to improve extraction efficiency. The concentration and purity of the extracted total genomic DNA were detected by NanoDrop ND2000 spectrophotometer (Thermo, USA), with the quality control criteria of OD260/OD280 between 1.8 and 2.0 and OD260/OD230 ≥1.8. The integrity of genomic DNA was verified by 1% agarose gel electrophoresis, and only DNA samples with clear bands (no obvious smearing) were used for subsequent experiments. A DNAse/RNase-Free ultrapure water was used as a negative control to exclude reagent and environmental contamination.

#### 2.6.2 DNA library construction and sequencing

Paired-end (PE) DNA library with an insert size of 350 bp was constructed for each sample according to previously described (Fang et al., 2018). The constructed metagenomic library was subjected to double quality control using Qubit fluorometer and Agilent 2100 Bioanalyzer. The library concentration was required to be ≥10 ng/μL, the fragment size distribution was concentrated between 300–500 bp, and the adapter contamination rate was < 1% to ensure the library quality met the sequencing requirements. The qualified libraries were sequenced on the BGI-EQ500 platform (BGI-Shenzhen, Shenzhen, China) using the PE150 paired-end sequencing strategy to generate raw sequencing reads.

#### 2.6.3 Data pre-processing

High-quality reads were obtained by filtering low-quality reads with ambiguous “N” bases>5% and low-quality base ratio >20% using Fastp v0.23.4 with parameters: -Q, --thread = 16, --length_required = 50, --n_base_limit = 2, --compression=6. Host DNA contamination was removed by mapping to GRCm39-mm39 genome reference consortium by bowtie2 v2.5.4 with parameters: -p 48, -x mm39_index, --un-conc-gz *RH.fq.gz, -S/dev/null. Over 90% of the raw reads were remained to be clean reads, and an average of 6 gigabytes of clean data were obtained for each sample.

#### 2.6.4 Taxonomic annotation and functional prediction

MetaPhlAn 4.0, which enables accurate taxonomic assignment at the phylum to species levels, was used to obtain the gastric microbial profiling,. Functional potential prediction of the microbial community was conducted via HUMAnN 4.0, which annotates functional genes and pathways based on the UniRef90 and KEGG databases.

#### 2.6.5 Diversity calculation

Alpha diversity (R 4.0.3 VEGAN: diversity (data, index = ‘richness/Shannon/Simpson/InSimpson’)) was calculated using the richness, Shannon, Simpson, and Inverse Simpson’s indexes of the taxonomic profiles. Beta diversity (R 4.0.3 APE: pcoa (‘bray_curtis distance’, correction = “none”, rn = NULL) between-sample diversity, R4.0.3 VEGAN: diversity (data, index = ‘bray_curtis distance’)) was calculated using the bray_curtis distance depending on the taxonomic profiles. PERMANOVA was performed on Bray-Curtis’s distance and 999 permutations in R (R 4.0.3: adonis (dist~phe, permutations = 1000)) to study the influencing factors of taxonomy between two groups.

#### 2.6.6 Differential analysis

Before differential analysis, we have firstly filtered out low-abundance and low-occurrence microbial species (mean relative abundance <0.001, occurrence rate <10%) to reduce sparsity during the differential analysis. Secondly we have used relative abundance with centered log-ratio (CLR) transformation to normalize the microbial abundance data, which effectively mitigates the compositional bias of microbiome data. The Wilcoxon rank-sum test was used to compare taxonomic composition (relative abundance of phyla/genera/species) and functional pathway enrichment between tumoral and peritumoral gastric microbiota samples, with statistical significance defined as P<0.05, as well as Effect size >0.1, mean occurrence >0.1. The false discovery rate (FDR) correction was used to correct the P-value.

### 2.7 Host-microbe interaction analysis

For the DEGs included in the correlation analysis, 7197 significantly differential DEGs (P<0.05 & |log_2_FC|>1) were identified by comparing 60 tumoral and 53 peritumoral tissues using Wilcoxon rank-sum test.

As for the significantly differential bacterial species included in the correlation analysis, we adopted a two-step screening strategy (1): First, we selected species with P<0.05 from Wilcoxon rank-sum test, and three bacterial species including *Serratia surfactantfaciens*, *Pseudomonas protegens*, *Selenomonas* sp. oral_taxon_126 were included (2). Additionally, we incorporated species meeting the criteria of mean occurrence >0.2, mean abundance >0.1, and effect size >0.1, and four additional species were further included whose P-value was between 0.1 and 0.2. For all P-values obtained from the Wilcoxon rank-sum test, we have adjusted them to Q-values using the Benjamini & Hochberg correction method, and these Q-values are presented in **Supplementary Tables 2A–D, 5C**.

Spearman’s rank correlation analysis was used to assess associations between significantly differentially abundant gastric bacterial species and significantly DEGs based on 60 tumoral and 53 peritumoral tissues that had both metagenomic and transccriptomic data. We have set a strict threshold for correlation analysis (Spearman’s ρ>0.18 or ρ<-0.18, P<0.05) and further filtered the results by functional enrichment (FDR <0.05) to ensure the biological significance of the correlations.

## 3 Results

### 3.1 Gene expression profiles between tumoral and peritumoral tissues

PERMANOVA was employed to assess the impact of clinical phenotypes on host gene expression. Lauren classification significantly influenced host gene expression in tumoral mucosa (P = 0.025), while *H. pylori* infection status was a significant factor in peritumoral mucosa (P = 0.0424) (**Table 1**).

**Table 1:** Results of PERMANOVA analyzing the influence of clinical phenotypes on gene expression profiles in tumoral and peritumoral tissues.

Phenotypes	Tumor			Peritumor		
	F.Model	R2	P-value	F.Model	R2	P-value
Sex	0.8431	0.0117	0.5475	1.3322	0.0184	0.2436
Age	1.1532	0.0160	0.2891	1.6475	0.0227	0.1546
Height	0.8475	0.0118	0.5482	0.6661	0.0093	0.5987
Weight	0.6718	0.0094	0.7411	1.1766	0.0163	0.2932
BMI	0.7921	0.0110	0.5978	1.0663	0.0148	0.3492
Chemotherapy	1.2782	0.0177	0.2147	0.9311	0.0129	0.4010
Cancerous site	0.9223	0.0257	0.5244	0.8305	0.0232	0.5546
Surgicalmethod	1.0637	0.0295	0.3555	1.7613	0.0479	0.0897
Tumor stage	1.4075	0.0387	0.1194	1.7691	0.0481	0.0894
**Lauren classification**	**1.6635**	**0.0674**	**0.0250**	1.5543	0.0633	0.1155
Differentiation degree	0.8592	0.0360	0.6524	0.8041	0.0338	0.6264
**Hp infection**	0.7794	0.0109	0.6186	**2.6324**	**0.0358**	**0.0424**
ECOG/CCI	0.8442	0.0118	0.5439	0.2740	0.0038	0.9250

Bold font indicates phenotypes with P<0.05

**Figure 2:**
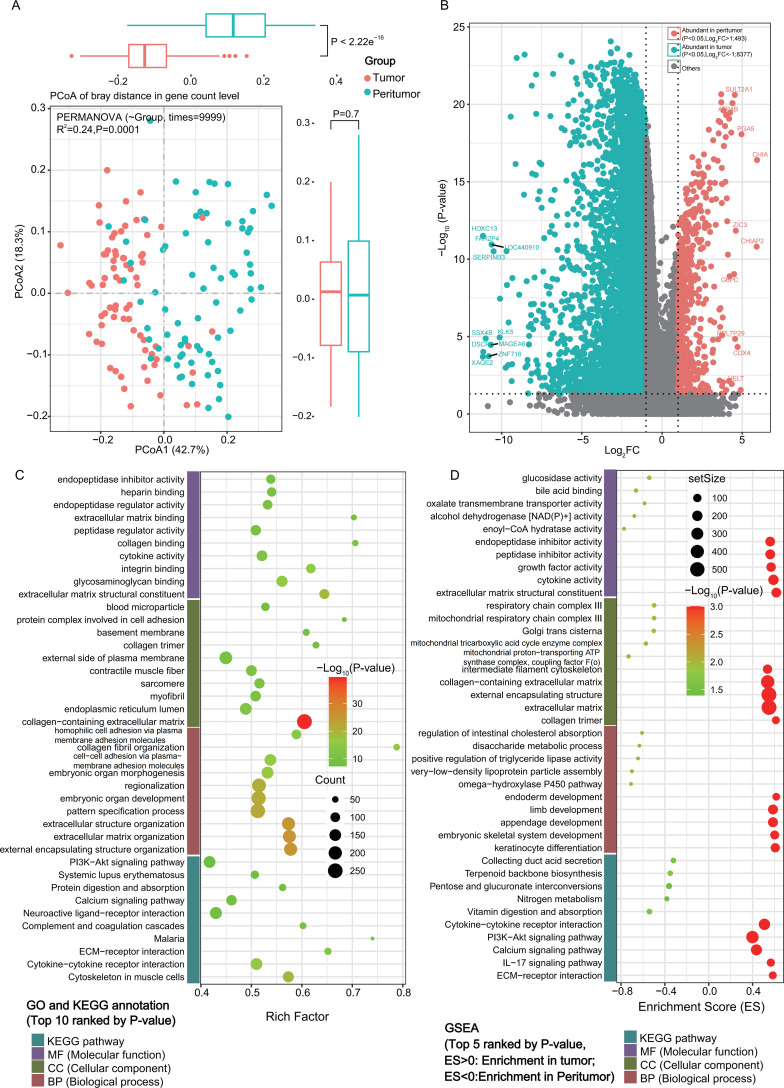
Transcriptomic profiling of 73 tumoral and 73 peritumoral mucosa from advanced gastric cancer (AGC) patients. **(A)** Principal coordinate analysis (PCoA) illustrating distinct clustering of transcriptomes from tumoral and peritumoral tissues (PERMANOVA, R^2^ = 0.24, P = 0.0001). **(B)** Heatmap and hierarchical clustering of 8870 differentially expressed genes (DEGs) between tumoral and peritumoral tissues. **(C)** The top 10 significantly enriched Gene Ontology (GO) terms and KEGG pathways based on analysis of the DEGs. **(D)** Gene set enrichment analysis (GSEA) identifying hallmark pathways dysregulated in tumoral and peritumoral tissues.

Transcriptomic analysis of 73 tumoral and 73 peritumoral tissues (58 with paired tumor and peritumoral tissues) from AGC patients detected 28224 genes across all samples (**Supplementary Table 2A**). PCoA revealed distinct gene expression profiles (**Figure 2A**, PERMANOVA, R²=0.24, P = 0.0001). Wilcoxon rank-sum test identified 19815 significantly different genes, with 18295 enriched in tumors and 1520 in peritumoral tissues (P<0.05, **Supplementary Table 2A**). Stricter threshold (P<0.05 and |Log_2_FC|>1) identified 8870 robust DEGs, with 8377 up-regulated in tumors and 493 in peritumoral tissues (**Figure 2B**). GSVA confirmed 8188 DEGs enriched in tumor (6375) and peritumoral (1803) regions (**Supplementary Table 2B**). Functional profiling of the DEGs was conducted using GO, KEGG, and GSEA (**Figure 2C**, **2D**; **Supplementary Tables 2C, D**).

GO analysis revealed DEGs associated with cellular component (CC) such as extracellular matrix (ECM), plasma membrane, cytoskeleton, and sarcomere; molecular function (MF) including oxidoreductase/cytokine/transmembrane transporter/ion channel activity, steroid/vitamin binding; and biological process (BP) related to digestion, lipid and hormone metabolism.

KEGG enrichment highlighted involvement in metabolic pathways including fat and protein digestion, bile secretion, steroid hormone biosynthesis, pyruvate metabolism, pentose and glucuronate interconversions, as well as PPAR signaling pathway and neuroactive ligand-receptor interactions.

GSEA further delineated tissue-specific signatures: in tumoral tissues, DEGs were enriched in ECM organization, cytokine activity, developmental processes, and oncogenic pathways such as PI3K-Akt and IL-17 signaling; in peritumoral tissues, DEGs were linked to mitochondrial and Golgi components, metabolic transport functions, and pathways including nitrogen metabolism, vitamin digestion, and terpenoid biosynthesis.

### 3.2 Hp infection modulates gene expression in peritumoral tissues

**Figure 3:**
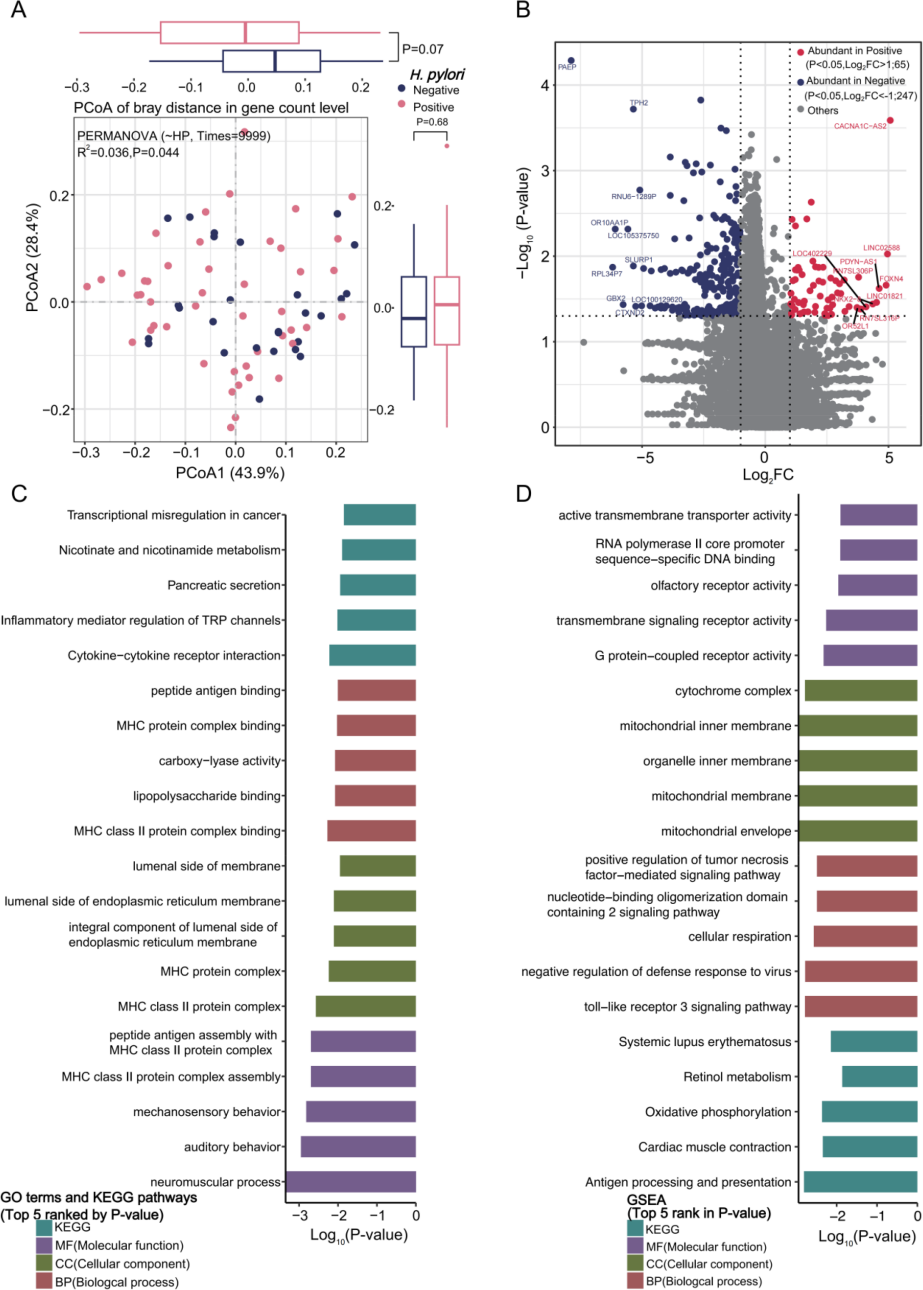
*Helicobacter pylori* (Hp) infection reprograms the transcriptomic landscape of peritumoral gastric tissues. **(A)** PCoA demonstrates distinct gene expression profiles between (H) pylori-positive and -negative peritumoral tissues. **(B)** Unsupervised hierarchical clustering of DEGs separates peritumoral samples based on Hp infection status. **(C)** Bar plot displaying the top five significantly enriched GO terms and KEGG pathways derived from the DEGs shown in panel **(B)**. **(D)** GSEA identifies key biological pathways significantly altered in Hp-positive compared to negative peritumoral tissues.

Consistent with PERMANOVA results, gene expression in peritumoral tissues differed between Hp-positive and Hp-negative tissues (**Figure 3A**, PCoA, n=73). A total of 2457 DEGs were identified (P<0.05, **Supplementary Table 3A**), with 65 significantly up-regulated in Hp-positive and 247 up-regulated in Hp-negative tissues under strict criteria (P<0.05, |Log_2_FC|>1; **Figure 3B**). GSVA confirmed the differences of genes between two groups (**Supplementary Table 3B**). Functional annotation revealed distinct pathway enrichment (**Figures 3C, D**; **Supplementary Tables 3C, D**).

GO terms included lumenal side of endoplasmic reticulum membrane, MHC protein complex (CC); peptide antigen assembly with MHC class II protein complex, mechanosensory behavior, auditory behavior, and neuromuscular process (MF); peptide antigen/lipopolysaccharide binding, MHC protein complex binding, and carboxy-lyase activity (BP).

KEGG pathways enriched in transcriptional misregulation in cancer, cytokine-cytokine receptor interaction, nicotinate/nicotinamide metabolism, and inflammatory mediator regulation of TRP channels.

GSEA highlighted mitochondrial components, immune signaling (e.g.,TNF, TLR3, NOD2 signaling pathway), oxidative phosphorylation, and antigen processing and presentation.

### 3.3 Lauren classification influences gene expression and function in tumor tissues

Per PERMANOVA results, Lauren classification significantly shaped gene expression in tumoral mucosa, with distinct transcriptomic and functional profiles identified across subtypes (27 intestinal-type, 32 diffuse-type, 20 mixed-type) (**Supplementary Table 4A**) and intergroup Demographic/clinical comparisons were shown in **Supplementary Table 1C**. The results showed that except for differentiation degree (χ²=36.00, P = 2.76e-06) and preoperative chemotherapy status (χ²=7.45, P = 0.024), no significant differences were observed among the demographic and clinical factors, which rules out the potential confounding of these factors on the subtype-specific gene expression and microbial-host interaction results.

#### 3.3.1 Diffuse- vs. intestinal-type

**Figure 4:**
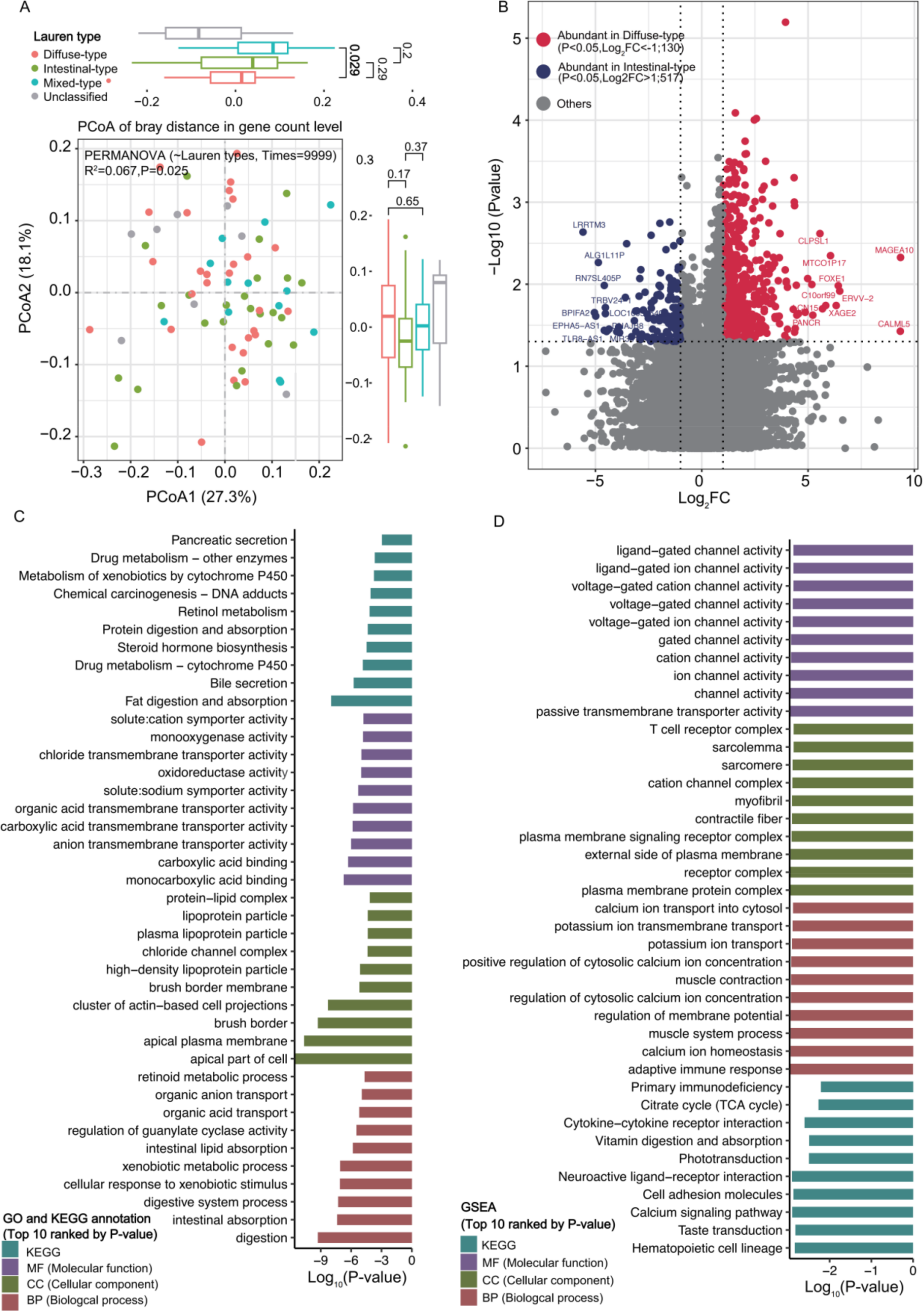
Transcriptional differences and functional enrichment in tumoral mucosa across Lauren classification subtypes. **(A)** PCoA reveals significant differences of gene expression profiles among intestinal-type, diffuse-type, mixed-type, and unclassified AGC subgroups (PERMANOVA, R^2^ = 0.067, P = 0.025). **(B)** Heatmap displaying DEGs between intestinal-type and diffuse-type AGC tissues. **(C)** The top 10 significantly enriched GO terms and KEGG pathways ranked by P-value. **(D)** The top 10 enriched gene sets from GSEA ranked by P-value.

PCoA demonstrated clear separation in gene expression (**Figure 4A**, PERMANOVA: P = 0.025, R^2^ = 0.067) and 1623 DEGs were identified between two group (Wilcoxon rank-sum test, P<0.05, **Supplementary Table 4A**). Among which 517 DEGs were up-regulated in intestinal-type while 130 were abundant in diffuse-type tissues under the criteria of P<0.05 and |Log_2_FC|>1 (**Figure 4B**).

Intestinal-type tumors (517 DEGs) were markedly enriched in metabolic functions, including nutrient digestion and absorption, xenobiotic metabolism, and ion transport (**Figure 4C**; **Supplementary Table 4B**). In contrast, diffuse-type tumors (130 DEGs) displayed distinct features. Both GO, KEGG pathways and GSEA consistently highlighted strong enrichment of immune and inflammatory pathways (e.g., adaptive immune response, cytokine signaling) and muscle/system processes (e.g., muscle contraction, calcium signaling) across the two subtypes (**Figures 4C, D**; **Supplementary Tables 4B, C**).

#### 3.3.2 Intestinal- vs. mixed-type

**Figure 5:**
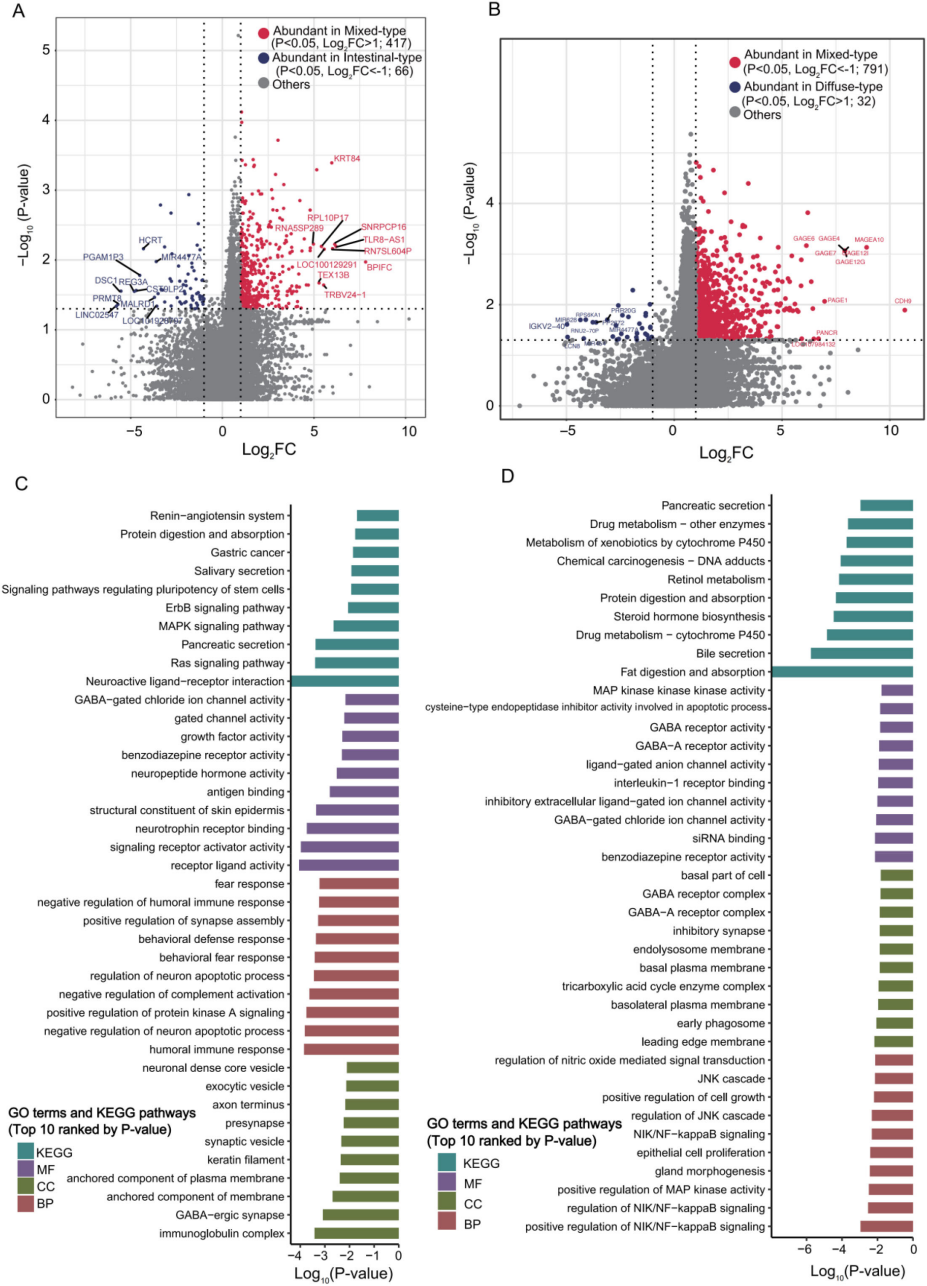
Transcriptional and functional differences among Lauren classification subtypes in AGC. **(A, B)** Heatmaps displaying the transcriptional profiles of DEGs in mixed-type tumors compared to intestinal-type **(A)** and diffuse-type **(B)** tumors. **(C, D)** Significantly enriched GO terms and KEGG pathways of the DEGs identified in the mixed-type versus intestinal-type **(C)** and mixed-type versus diffuse-type **(D)** comparisons.

**Figure 6:**
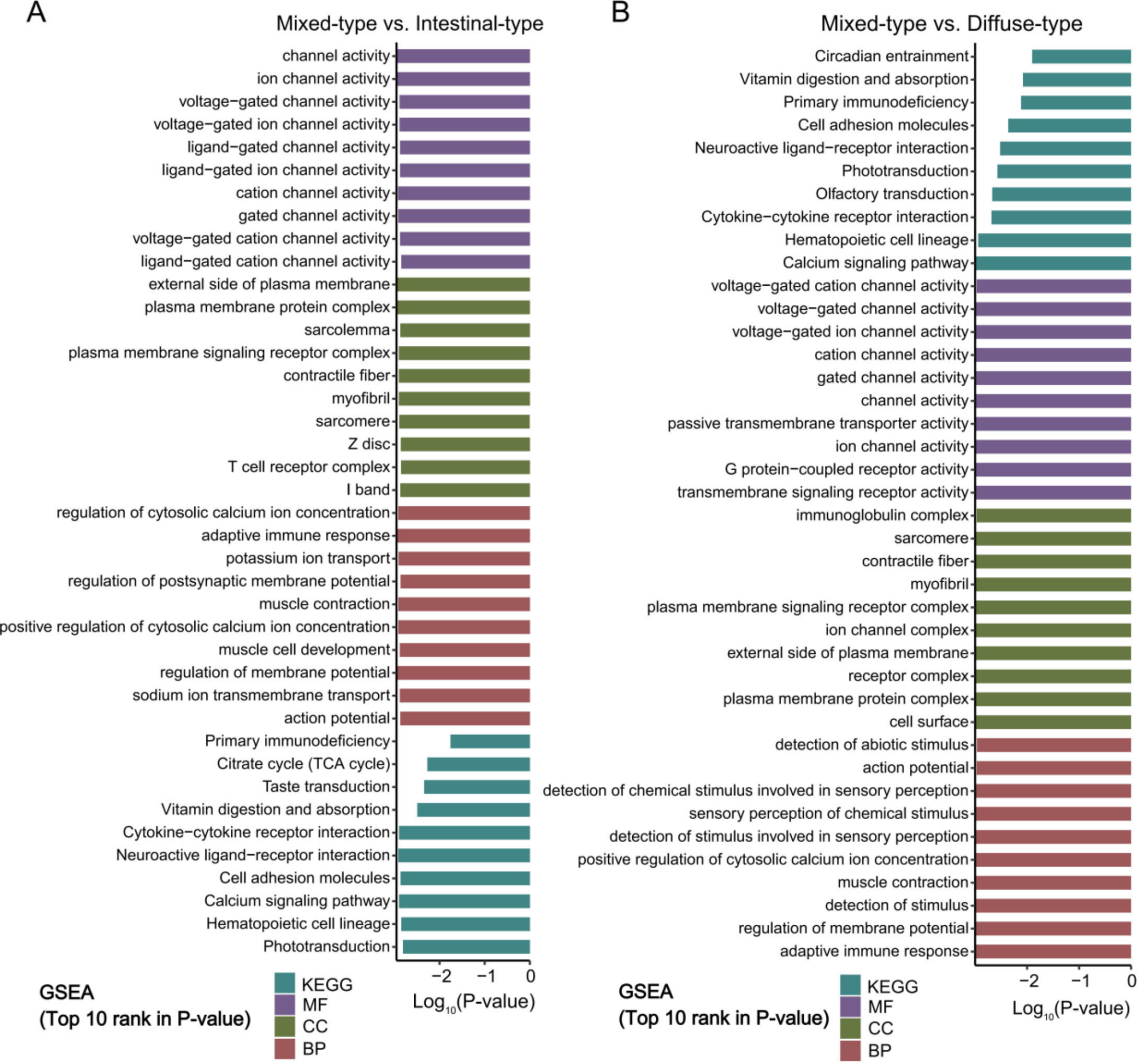
Fucntional enrichment analysis comparing mixed-type versus other two AGC subtypes. Top 10 most significantly enriched GO terms and KEGG pathways (ranked by P-value) from GSEA for the comparisons between mixed-type and intestinal-type **(A)**, and mixed-type and diffuse-type **(B)** AGC.

Differential analysis identified 1867 DEGs (**Supplementary Table 4A**), with 417 significantly up-regulated in mixed-type, while 66 DEGs in intestinal-type tissues with stricter criteria (**Figure 5A**, P<0.05, |Log_2_FC|>1). Functional profiling revealed marked subtype specificity using GO, KEGG, and GSEA (**Figures 5C**, **6A**; **Supplementary Tables 4D, E**).

Mixed-type enriched DEGs were associated with immune signaling (e.g., humoral response, antigen binding) and receptor activity (e.g., neuropeptide hormone, receptor activator), linked to pathways like Ras/MAPK signaling and neuroactive ligand-receptor interaction.

Intestinal-type DEGs were related to structural components (e.g., myofibril, sarcomere) and ion channel/transport activity, enriched in metabolic pathways including the TCA cycle and vitamin digestion/absorption.

#### 3.3.3 Diffuse- vs. mixed-type

Comparative transcriptomic analysis identified 4610 DEGs between two groups, with 791 DEGs significantly enriched in mixed-type and 32 in diffuse-type tumors (**Figure 5B**, P<0.05, |Log_2_FC|>1). Functional analysis indicate pronounced differences in immune signaling and metabolic processes between the two subtypes (**Figures 5D**, **6B**; **Supplementary Tables 4F, G**), details as below:

DEGs were associated with GO terms including immune and synaptic structures (e.g., inhibitory synapse, GABA receptor), and biological processes including NF-κB and MAPK signaling, epithelial proliferation, and gland morphogenesis.

Enriched KEGG pathways included protein digestion and absorption, bile secretion, steroid hormone biosynthesis, and xenobiotic metabolism.

GSEA highlighted enrichment in immune response, ion channel activity, G protein-coupled receptor signaling, calcium regulation, and pathways such as cytokine-receptor interaction and cell adhesion in mixed-type tumors.

### 3.4 Host-microbe interactions

Integrated analysis of 53 peritumoral and 60 tumoral mucosal samples with paired transcriptomic and metagenomic data (**Supplementary Table 5A**) identified seven key bacterial species (**Supplementary Table 5C**) for correlation studies. Spearman’s rank correlation analysis between these seven bacterial species (**Supplementary Table 5C**) and 8870 DEGs (**Supplementary Table 5D**) revealed 13199 significant associations (**Supplementary Table 5E**). The number of DEGs correlated with each species varied substantially: *S. surfactantfaciens* (6465), *P. protegens* (4417), *Selenomonas* sp (853). *H. pylori* (831), *Alloprevotella* sp (289)., *P. jejuni* (207), and *S. infantis* (137). Functional enrichment analysis of these correlated DEGs identified distinct biological pathways associated with each bacterial species. We mainly focus on *Serratia surfactantfaciens* and *Pseudomonas protegens*, as they were the only ones that met both screening criteria simultaneously.

#### 3.4.1 Tumor-enriched species (S. surfactantfaciens, P. protegens)

**Figure 7:**
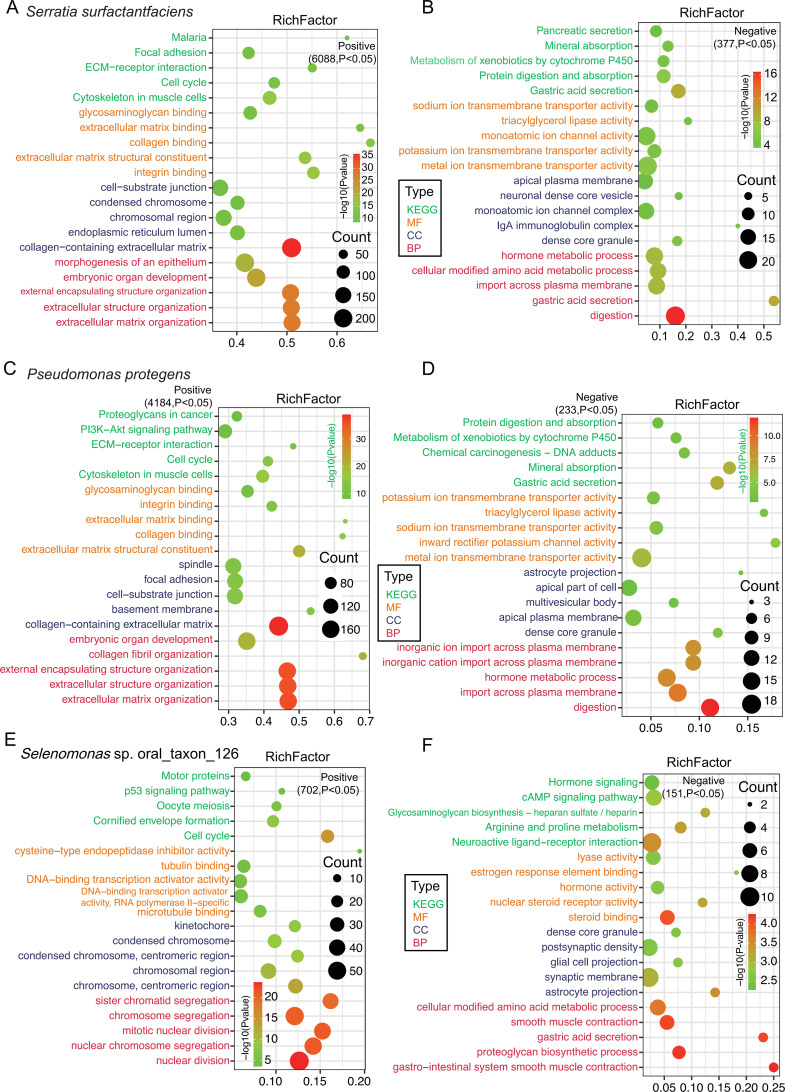
Fucntional enrichment analysis comparing mixed-type versus other two AGC subtypes. Top 10 most significantly enriched GO terms and KEGG pathways (ranked by P-value) from GSEA for the comparisons between mixed-type and intestinal-type **(A)**, and mixed-type and diffuse-type **(B)** AGC.

6088 DEGs were positively while 1107 were negatively correlated with *S. surfactantfaciens.* 4184 were positively and 233 were negatively associated with *P. protegens.* The positively correlated DEGs were mainly involved in tumor-promoting pathways, including ECM/collagen fibril organization, ECM-receptor interaction, cell cycle regulation, PI3K-Akt signaling, focal adhesion, epithelium morphogenesis, and proteoglycans in cancer (**Figures 7A, C**; **Supplementary Tables 5H, F**). Negatively correlated DEGs were primarily linked to metabolic processes like protein digestion and xenobiotic metabolism. (**Figures 7B, D**; **Supplementary Tables 5G, I**).

#### 3.4.2 Peritumoral-enriched *Selenomonas* sp. oral_taxon_126

This species showed positive associations with 702 DEGs and negative correlations with 151 DEGs. Positively associated DEGs involved in microtubule/tubulin binding modulation, p53 signaling pathway, cell cycle and mitotic processes (**Figure 7E**, **Supplementary Table 5J**). Negatively associated DEGs were involved in metabolic processes like gastrointestinal smooth muscle contraction, proteoglycan/glycosaminoglycan biosynthesis, gastric acid secretion, arginine/proline metabolism, cAMP/hormone signaling (**Figure 7F**, **Supplementary Table 5K**).

#### 3.4.3 DEGs associated with *H. pylori*

*H. pylori* showed a positive correlation with 721 DEGs enriched in immune pathways (e.g., lymphocyte-mediated immunity, cytokine-cytokine receptor interaction) (**Supplementary Figure 1A**, **Supplementary Table 5L**), whereas it was negatively correlated with 110 DEGs involved in metabolic processes like cilium movement and mineral absorption (**Supplementary Figure 1B**, **Supplementary Table 5M**).

#### 3.4.4 Other species exhibited distinct correlation patterns

*Alloprevotella* sp. oral_taxon_473: Positively correlated DEGs (224) were linked to keratinocyte differentiation and cancer transcriptional misregulation (**Supplementary Figure 1C**, **Supplementary Table 5N**); negatively correlated DEGs (65) were involved in T-cell receptor signaling, antigen-mediated signaling, and NK cell cytotoxicity (**Supplementary Figure 1D**, **Supplementary Table 5O**).

*P. Jejuni*: Positively correlated DEGs (97) participated in chemokine production, synaptic and muscle-related functions (**Supplementary Figure 1E**, **Supplementary Table 5P**); negatively correlated DEGs (110) were associated with mesenchymal apoptosis and vitamin metabolism (**Supplementary Figure 1F**, **Supplementary Table 5Q**).

*S. infantis*: Positively correlated DEGs (76) were enriched in neuroactive ligand-receptor interaction, complement/coagulation cascades, and PPAR signaling (**Supplementary Figure 1G**, **Supplementary Table 5R**); negatively correlated DEGs (61) functioned in columnar/cuboidal/neuro epithelial cell differentiation and hormone signaling pathways (**Supplementary Figure 1H**, **Supplementary Table 5S**).

## 4 Discussion

AGC remains a major global health burden, with its pathogenesis involving complex interactions among host genetics, environmental factors, and the gastric microbiota (Sundar et al., 2025). In this study, we integrated transcriptomic and metagenomic data to investigate the dynamic relationships between clinical phenotypes, host gene expression, and gastric microbes in AGC patients from Northwestern China, providing novel insights into the molecular mechanisms driving AGC development and progression, and offers valuable implications for regional precision medicine targeting AGC.

### 4.1 Clinical phenotypes as modulators of host gene expression

A key finding of this study is that clinical phenotypes—specifically Lauren classification and Hp infection—exert distinct regulatory effects on host gene expression in tumoral and peritumoral tissues. Lauren classification significantly shaped gene expression profiles in tumoral mucosa (P = 0.025), while Hp infection primarily influenced gene expression in peritumoral mucosa (P = 0.0424). Lauren classification tumour subtypes predicted survival and responded differently to chemotherapy (Jimenez et al., 2017). Hp induces chronic inflammation affecting the gastric epithelium, which can lead to DNA damage and the promotion of precancerous lesions (Reyes, 2023). This tissue-specific regulation aligns with the distinct biological roles of tumoral and peritumoral tissues: tumoral tissues represent the neoplastic core, where histological subtypes (e.g., intestinal vs. diffuse) drive divergent oncogenic pathways, whereas peritumoral tissues serve as a “microenvironmental buffer” sensitive to microbial perturbations like Hp infection (Zhang et al., 2023).

The demographic and clinical characteristics of our cohort (88 AGC patients, predominantly male [76/88], mean age 59.01 ± 9.23 years) are consistent with global epidemiological trends of AGC, which shows higher incidence in middle-aged and elderly males (Sundar et al., 2025). The retention of samples with genus-level Shannon index >0.1 ensured high-quality microbial data, while the inclusion of paired transcriptomic-metagenomic samples (60 tumoral, 53 peritumoral) enhanced the reliability of correlation analyses.

### 4.2 Tumoral and peritumoral gene expression: significant divergence

PCoA clearly distinguished gene expression profiles between tumoral and peritumoral tissues (PERMANOVA, R²=0.24, P = 0.0001), with 8870 DEGs identified under strict criteria (P<0.05, |Log_2_FC|>1). The overwhelming majority of DEGs (8377/8870) were up-regulated in tumoral tissues, reflecting the profound transcriptional reprogramming associated with malignant transformation. Excluding sequencing potential mistakes (**Supplementary Figure 2**), This phenomena is consistent with published gastric cancer transcriptomic studies (Totoki et al., 2023), which also reported a high proportion of upregulated genes in GC tumor tissues (85-95%) compared with adjacent normal tissues. In another gastric cancer and adjacent normal tissue study performed by Cicek et al (Balikci and Kucukakcali, 2025), they have identified 627 significantly differentially expressed genes (DEGs) between cancer and AN tissues, comprising 201 upregulated and 426 downregulated genes (|log_2_FC| > 1, adjusted p-value < 0.05), which might be due to the inequality between the two groups of samples (96 GC samples vs. 12 adjacent normal tissues).

Functional annotation highlighted that tumoral DEGs were enriched in pathways critical for cancer progression: ECM organization, ECM-receptor interaction, focal adhesion, PI3K-Akt pathway and cell cycle regulation. These pathways are well-documented drivers of AGC invasiveness and metastasis. ECM organization and remodeling is a hallmark of gastric cancer invasion and metastasis, and the upregulation of ECM-related genes (e.g., COL1A1, FN1, ITGA5) in tumor tissues promotes the degradation of the basement membrane and tumor cell migration (Mai et al., 2024; Prakash and Shaked, 2024), facilitates tumor cell migration (Mai et al., 2024; Prakash and Shaked, 2024). The PI3K-Akt pathway is one of the most frequently activated oncogenic pathways in AGC, and its activation promotes uncontrolled cell proliferation, anti-apoptosis, and angiogenesis (Fattahi et al., 2020; Lei ZN. et al., 2022), while dysregulated cell cycle control promotes uncontrolled proliferation (Vermeulen et al., 2003). Further analysis of the crosstalk between ECM organization and PI3K-Akt signaling found that 89% of ECM-related DEGs are upstream regulators of the PI3K-Akt pathway (e.g., integrin family genes ITGA5/ITGB1). This is consistent with the known mechanism that ECM remodeling activates the PI3K-Akt pathway to promote gastric cancer progression (Mai et al., 2024), and further clarifies the functional link between the enriched pathways in our study. Conversely, peritumoral DEGs were enriched in metabolic pathways (e.g., retinol metabolism, bile secretion) and mitochondrial functions (e.g., respiratory chain complex III), consistent with the role of peritumoral tissues in maintaining physiological homeostasis and potentially restraining tumor expansion (Koca et al., 2024).

GSEA further reinforced these differences: tumoral tissues showed enrichment in endoderm development and keratinocyte differentiation (processes linked to epithelial-mesenchymal transition (Yan et al., 2024), EMT), while peritumoral tissues were enriched in cholesterol absorption and triglyceride metabolism (key for maintaining mucosal barrier function (Cui et al., 2022; Xiao et al., 2023)). These findings align with previous studies showing that tumoral tissues prioritize oncogenic processes over metabolism, whereas peritumoral tissues retain residual physiological functions (de Visser and Joyce, 2023; Altea-Manzano et al., 2025; Kay and Zanivan, 2025).

### 4.3 Hp infection: a key regulator of peritumoral immune and metabolic pathways

Hp infection is a well-established risk factor for GC, but its tissue-specific effects on gene expression in AGC remain understudied. Our results showed that Hp-positive peritumoral tissues exhibited 65 up-regulated and 247 down-regulated DEGs, with functional enrichment in immune responses (e.g., antigen processing and presentation via MHC class II), inflammatory signaling (TLR3 and NOD2 pathways), and mitochondrial metabolism (oxidative phosphorylation).

The enrichment of MHC class II-related DEGs in Hp-positive peritumoral tissues suggests that Hp infection triggers adaptive immune activation in the peritumoral microenvironment—potentially a compensatory response to microbial-induced tissue damage (Moyat and Velin, 2014; Takayanagi et al., 2025). However, the concurrent up-regulation of inflammatory pathways (e.g., TLR3 signaling) may paradoxically promote carcinogenesis, as chronic inflammation is a known driver of AGC (Jaroenlapnopparat et al., 2022). Additionally, the down-regulation of oxidative phosphorylation-related genes in Hp-positive tissues could reflect microbial-induced mitochondrial dysfunction, which has been linked to gastric epithelial cell apoptosis resistance and malignant transformation (Foo et al., 2022; Han et al., 2022).

Notably, Hp infection did not significantly affect tumoral gene expression, which may be due to the fact that tumoral tissues have already undergone irreversible oncogenic transformation (Massey et al., 2024), rendering them less sensitive to microbial perturbations. This finding underscores the importance of targeting peritumoral tissues in Hp-related AGC intervention strategies.

### 4.4 Lauren classification as a determinant of intrinsic tumor biology

Lauren classification (intestinal-, diffuse-, mixed-types) is a critical prognostic marker for AGC (Jimenez et al., 2017), and our study revealed subtype-specific gene expression patterns that shed light on their distinct biological behaviors.

#### 4.4.1 Diffuse-type vs. Intestinal-type

Diffuse-type AGC is characterized by infiltrative growth and poor prognosis, while intestinal-type AGC is associated with a more indolent course (Jimenez et al., 2017). Our results showed that intestinal-type tumoral tissues had 517 up-regulated DEGs enriched in nutrient metabolism (protein/fat digestion, bile secretion) and adaptive immune responses (T cell receptor signaling), whereas diffuse-type tissues had 130 up-regulated DEGs linked to calcium signaling and muscle contraction. These differences may explain the distinct clinical phenotypes: intestinal-type AGC’s reliance on metabolic pathways supports its glandular growth pattern (Wang et al., 2021; Wu et al., 2024), while diffuse-type AGC’s enrichment in calcium signaling (a driver of cell motility) facilitates its infiltrative behavior (Giorgi et al., 2018).

#### 4.4.2 Mixed-type vs. Intestinal/Diffuse-types

Mixed-type AGC, a heterogeneous subtype with variable prognosis, showed the most distinct gene expression profile: 417 up-regulated DEGs compared to intestinal-type (enriched in Ras/MAPK signaling and stem cell pluripotency pathways) and 791 up-regulated DEGs compared to diffuse-type (enriched in NF-κB signaling and adaptive immunity). The enrichment of Ras/MAPK signaling—an oncogenic pathway frequently mutated in AGC (Bahar et al., 2023)—suggests mixed-type AGC may have higher aggressiveness, while its enhanced immune response could imply potential sensitivity to immunotherapy. These findings highlight the need for subtype-specific treatment strategies for AGC.

### 4.5 Gastric microbiota-host interactions: uncovering functional synergies

A particularly novel aspect of our study is the detailed correlation analysis between specific gastric microbiota members and host DEGs, which revealed an extensive network of associations extending far beyond *H. pylori*. The identification of over 13,000 significant correlations underscores the pervasive influence of the microbiota on the host transcriptome. The species-specific correlation patterns suggest distinct mechanistic roles for different bacteria in shaping the TME.

#### 4.5.1 Pro-tumorigenic candidates: *S. surfactantfaciens* and *P. protegens*

*S. surfactantfaciens* and *P. protegens*, both enriched in tumoral tissues, correlated with 6465 and 4417 DEGs, which seems a risk of overfitting. We have divided the samples into Lauren subtype subgroups (intestinal, diffuse, mixed) and Hp infection subgroups, and found that the core correlations between *S. surfactantfaciens* and ECM/cell cycle-related genes (e.g., MTHFD1L, CRABP2, ITGA5) were consistent across all subgroups (Spearman’s ρ>0.3, P<0.01), indicating that these correlations are not driven by a single subgroup. Then we performed 1000 times bootstrap resampling (randomly sampling 80% of the samples each time) on the correlation data, and the results showed that 92.3% of the correlations between *S. surfactantfaciens* and host genes were consistently significant (P<0.05) in more than 90% of the resampling iterations, which is far higher than the random level (5%), confirming the robustness of the correlations.

For the functional clustering analysis, both *S. surfactantfaciens* and *P. protegens* showed strong positive correlations with DEGs involved in a small number of core oncogenic pathways, suhc as ECM remodeling (e.g., *MTHFD1L*, *CRABP2*), focal adhension, the PI3K-Akt pathway, and cell cycle regulation, rather than randomly distributed across all pathways, which indicates that the large number of correlated genes is due to the global regulation of core oncogenic pathways by *S. surfactantfaciens* and *P. protegens* rather than random overfitting. Furthermore, the core genes correlated with *S. surfactantfaciens* and *P. protegens* (e.g., ECM-related genes ITGA5, COL1A1, FN1) are well-documented oncogenes in gastric cancer (Zhu et al., 2021; Mai et al., 2024; Prakash and Shaked, 2024), and their upregulation is closely associated with GC progression, supports the biological significance of the correlations. These results suggest *S. surfactantfaciens* and *P. protegens* as potential pro-tumorigenic actors, represents a paradigm-shifting finding, implicating non-*H. pylori* species in directly influencing pathways central to cancer progression, aligns with previous studies linking these microbes to biofilm formation and epithelial cell invasion (Choi et al., 2023), suggesting they may promote AGC progression by enhancing tumor cell motility and proliferation. Conversely, their negative correlation with metabolic DEGs (e.g., *ADIPOQ*, *ADH7*) hinting that these microbes might be associated with, or contribute to, the suppression of normal gastric function, which further supports the idea that these microbes disrupt mucosal metabolism to favor tumor growth.

#### 4.5.2 The dual role of *H. pylori* in correlation analysis

*H. pylori*, though only slightly more abundant in peritumoral tissues, showed positive correlations with DEGs localized to immunoglobulin complexes and involved in leukocyte-mediated immunity (e.g., *JAK3*, *LAG3*), lymphocyte activity, and chemokine signaling, reinforcing its role as an immune activator. The negative correlations with DEGs involved in specialized functions like cilium movement and mineral absorption add nuance, suggesting that chronic *H. pylori* infection may also lead to the loss of certain epithelial functions.

#### 4.5.3 Immunomodulatory and niche-specific effects of other taxa

Except for Hp, other bacterial species displayed more specialized associations. *Alloprevotella* sp. oral_taxon_473 was notably linked to immune pathways, with its negative correlation with T-cell receptor signaling and Th cell differentiation suggesting a potential immunosuppressive role. In contrast, *Selenomonas* sp. oral_taxon_126—enriched in peritumoral tissues—correlated with DEGs involved in cell cycle regulation, mitotic processes, p53 signaling (a tumor suppressor pathway), suggesting it may directly or indirectly influence host cell proliferation and genomic integrity, exert a protective effect by enhancing cell cycle checkpoints, a potential that requires functional validation.

#### 4.5.4 Metabolic regulators: *P. jejuni* and *S. infantis*

*P. jejuni* and *S. infantis* showed correlations with DEGs involved in hormone metabolism (e.g., thyroid hormone synthesis) and neuroactive ligand-receptor interaction, respectively. Additionally, *S. infantis* was associated with both immune (complement activation) and metabolic (PPAR signaling) pathways, indicating multifaceted interactions. These findings suggest *P. jejuni* and *S. infantis* may modulate AGC pathogenesis by disrupting mucosal endocrine function—a previously understudied mechanism.

Our study provides novel insights into the role of gastric bacteria in gastric cancer among AGC patients from Northwestern China. However, current research relies predominantly on multi-omics data, bioinformatic analysis using known tools (e.g., HumanN4.0 for function prediction) and correlation analyses, highlighting the necessity of validation experiments to confirm the identified potential targets and pathways. These experiments will include (1): perform *de novo* assembly of the gastric microbial metagenome to identify novel genes and functional pathways (2); *in vitro* cell-microbe co-culture assays combined with qRT-PCR, Western blot, CCK-8, and Transwell analyses to verify direct microbial regulation of gastric cancer cells (3); microbial gene knockout experiments to assess attenuation or abrogation of this regulatory effect (4); *in vivo* validation using gastric cancer PDX mouse models colonized with *S. surfactantfaciens*, focusing on tumor growth, metastasis, and host gene expression to confirm pro-tumorigenic activity; and (5) multicenter clinical validation in Northwest China to correlate *S. surfactantfaciens/P. protegens* abundance with ECM-related gene expression and clinical prognosis (overall survival, progression-free survival), thereby confirming clinical relevance.

### 4.6 Limitations and future directions

Several limitations existed (1): this cross-sectional design prevents causal inference between microbiota and gene expression (2); the small sample size may limit generalizability (3); the lack of functional validation (e.g., *in vitro* experiments) means the observed correlations cannot be confirmed as causal. Future studies should use longitudinal designs, larger cohorts, and functional assays to validate these findings. Additionally, metabolomic data (e.g., LC-MS/MS for gastric mucosal metabolites) should be used to validate the actual activity of microbial metabolic pathways and confirm the functional crosstalk between microbes and host metabolism.

## 5 Conclusions

This integrated analysis demonstrates that clinical phenotypes (Lauren classification, *H. pylori* infection) and the gastric microbiota synergistically modulate host gene expression in advanced gastric cancer (AGC) from Northwestern China, with tissue-specific effects on oncogenic, immune, and metabolic pathways. The identified microbiota-host correlations proposed potential therapeutic targets (e.g., targeting *S. surfactantfaciens* to inhibit ECM remodeling or leveraging *Selenomonas* sp. oral_taxon_126 to enhance p53 signaling) have better applicability in the Northwest Chinese population. However, further validation experiments are therefore warranted to confirm the functional relevance of these candidate targets and pathways. Collectively, these findings deepen our mechanistic understanding of AGC pathogenesis and lay a foundation for the development of personalized diagnostic and therapeutic strategies.

## Data Availability

The datasets presented in this study can be found in online repositories. The names of the repository/repositories and accession number(s) can be found below: https://www.ncbi.nlm.nih.gov/, PRJNA1067082 https://www.ncbi.nlm.nih.gov/, PRJNA1013242.
