# Supplementary material for: Integrated multi-omics profiling reveals phenotype- and tissue-specific host-microbiota interactions in paired tumor and peritumoral tissues of advanced gastric cancer patients from Northwest China
Source: Front Cell Infect Microbiol. 2026 Apr 20;16:1763765. doi: 10.3389/fcimb.2026.1763765 (PMC13136179; doi:10.3389/fcimb.2026.1763765)

Supplementary Figure 1. Functional enrichment of DEGs associated with differential gastric bacterial species. (**A, B**) Top five enriched GO terms and KEGG pathways for DEGs positively (A) and negatively (B) correlated with *Helicobacter pylori*. (**C, D**) Top five GO terms and KEGG pathways for DEGs significantly positively (C) and negatively (D) correlated with *Alloprevotella* sp. oral_taxon_473, involving cell cycle regulation and substance metabolism. (**E, F**) Top five GO terms and KEGG pathways for DEGs positively (E) and negatively (F) correlated with *Prevotella jejuni*. (**G, H**) Top five GO terms and KEGG pathways for DEGs positively (G) and negatively (H) correlated with *Streptococcus infantis*.

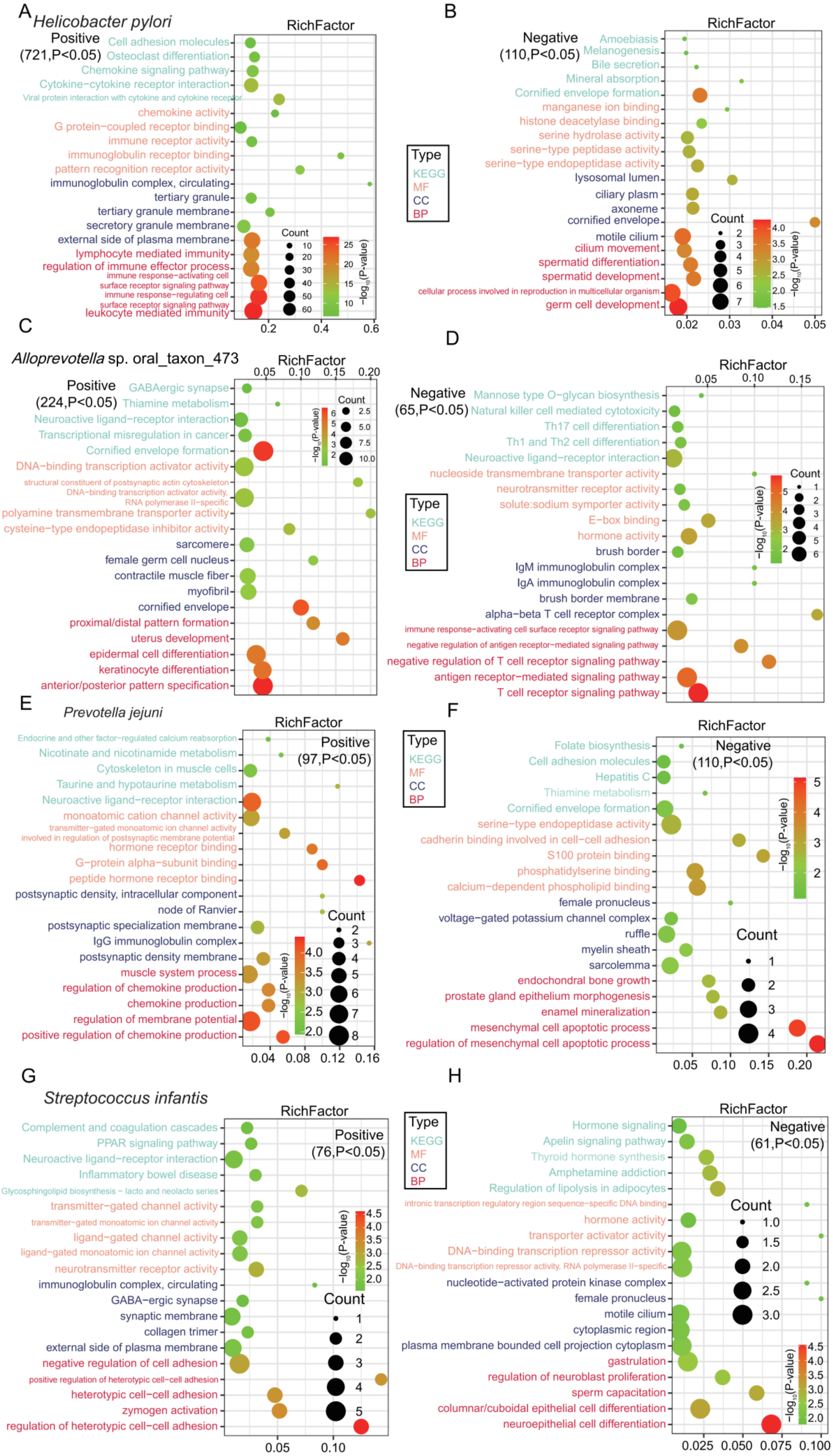

A
Helicobacter pylori
Positive
(721,P<0.05)
RichFactor
Cell adhesion molecules
Osteoclast differentiation
Chemokine signaling pathway
Cytokine−cytokine receptor interaction
Viral protein interaction with cytokine and cytokine receptor
chemokine activity
G protein−coupled receptor binding
immune receptor activity
immunoglobulin receptor binding
pattern recognition receptor activity
immunoglobulin complex, circulating
tertiary granule
tertiary granule membrane
secretory granule membrane
external side of plasma membrane
lymphocyte mediated immunity
regulation of immune effector process
immune response−activating cell surface receptor signaling pathway
immune response−regulating cell surface receptor signaling pathway
leukocyte mediated immunity
Count
10
20
30
40
50
60
−log10(P-value)
25
20
15
10
0.2
0.4
0.6
B
Negative
(110,P<0.05)
RichFactor
Amoebiasis
Melanogenesis
Bile secretion
Mineral absorption
Cornified envelope formation
manganese ion binding
histone deacetylase binding
serine hydrolase activity
serine−type peptidase activity
serine−type endopeptidase activity
lysosomal lumen
ciliary plasm
axoneme
cornified envelope
motile cilium
cilium movement
spermatid differentiation
spermatid development
cellular process involved in reproduction in multicellular organism
germ cell development
Type
KEGG
MF
CC
BP
Count
2
3
4
5
6
7
4.0
3.5
3.0
2.5
2.0
1.5
−log10(P-value)
0.02
0.03
0.04
0.05
C
Alloprevotella sp. oral_taxon_473
Positive
(224,P<0.05)
RichFactor
0.05
0.10
0.15
0.20
GABAergic synapse
Thiamine metabolism
Neuroactive ligand−receptor interaction
Transcriptional misregulation in cancer
Cornified envelope formation
DNA−binding transcription activator activity
structural constituent of postsynaptic actin cytoskeleton
DNA−binding transcription activator activity, RNA polymerase II−specific
polyamine transmembrane transporter activity
cysteine−type endopeptidase inhibitor activity
sarcomere
female germ cell nucleus
contractile muscle fiber
myofibril
cornified envelope
proximal/distal pattern formation
uterus development
epidermal cell differentiation
keratinocyte differentiation
anterior/posterior pattern specification
Count
2.5
5.0
7.5
10.0
−log10(P-value)
6
5
4
3
2
D
Negative
(65,P<0.05)
RichFactor
0.05
0.10
0.15
Mannose type O−glycan biosynthesis
Natural killer cell mediated cytotoxicity
Th17 cell differentiation
Th1 and Th2 cell differentiation
Neuroactive ligand−receptor interaction
nucleoside transmembrane transporter activity
neurotransmitter receptor activity
solute:sodium symporter activity
E−box binding
hormone activity
brush border
IgM immunoglobulin complex
IgA immunoglobulin complex
brush border membrane
alpha−beta T cell receptor complex
immune response−activating cell surface receptor signaling pathway
negative regulation of antigen receptor−mediated signaling pathway
negative regulation of T cell receptor signaling pathway
antigen receptor−mediated signaling pathway
T cell receptor signaling pathway
Type
KEGG
MF
CC
BP
−log10(P-value)
5
4
3
2
Count
1
2
3
4
5
6
E
Prevotella jejuni
RichFactor
Positive
(97,P<0.05)
Endocrine and other factor−regulated calcium reabsorption
Nicotinate and nicotinamide metabolism
Cytoskeleton in muscle cells
Taurine and hypotaurine metabolism
Neuroactive ligand−receptor interaction
monoatomic cation channel activity
transmitter−gated monoatomic ion channel activity involved in regulation of postsynaptic membrane potential
hormone receptor binding
G−protein alpha−subunit binding
peptide hormone receptor binding
postsynaptic density, intracellular component
node of Ranvier
postsynaptic specialization membrane
IgG immunoglobulin complex
postsynaptic density membrane
muscle system process
regulation of chemokine production
chemokine production
regulation of membrane potential
positive regulation of chemokine production
Count
2
3
4
5
6
7
8
−log10(P-value)
4.0
3.5
3.0
2.5
2.0
0.04
0.08
0.12
0.16
F
Type
KEGG
MF
CC
BP
RichFactor
Negative
(110,P<0.05)
Folate biosynthesis
Cell adhesion molecules
Hepatitis C
Thiamine metabolism
Cornified envelope formation
serine−type endopeptidase activity
cadherin binding involved in cell−cell adhesion
S100 protein binding
phosphatidylserine binding
calcium−dependent phospholipid binding
female pronucleus
voltage−gated potassium channel complex
ruffle
myelin sheath
sarcolemma
endochondral bone growth
prostate gland epithelium morphogenesis
enamel mineralization
mesenchymal cell apoptotic process
regulation of mesenchymal cell apoptotic process
−log10(P-value)
5
4
3
2
Count
1
2
3
4
0.05
0.10
0.15
0.20
G
Streptococcus infantis
RichFactor
Positive
(76,P<0.05)
Complement and coagulation cascades
PPAR signaling pathway
Neuroactive ligand−receptor interaction
Inflammatory bowel disease
Glycosphingolipid biosynthesis − lacto and neolacto series
transmitter−gated channel activity
transmitter−gated monoatomic ion channel activity
ligand−gated channel activity
ligand−gated monoatomic ion channel activity
neurotransmitter receptor activity
immunoglobulin complex, circulating
GABA−ergic synapse
synaptic membrane
collagen trimer
external side of plasma membrane
negative regulation of cell adhesion
positive regulation of heterotypic cell−cell adhesion
heterotypic cell−cell adhesion
zymogen activation
regulation of heterotypic cell−cell adhesion
−log10(P-value)
4.5
4.0
3.5
3.0
2.5
2.0
Count
1
2
3
4
5
0.05
0.10
H
Type
KEGG
MF
CC
BP
RichFactor
Negative
(61,P<0.05)
Hormone signaling
Apelin signaling pathway
Thyroid hormone synthesis
Amphetamine addiction
Regulation of lipolysis in adipocytes
intronic transcription regulatory region sequence−specific DNA binding
hormone activity
transporter activator activity
DNA−binding transcription repressor activity
DNA−binding transcription repressor activity, RNA polymerase II−specific
nucleotide−activated protein kinase complex
female pronucleus
motile cilium
cytoplasmic region
plasma membrane bounded cell projection cytoplasm
gastrulation
regulation of neuroblast proliferation
sperm capacitation
columnar/cuboidal epithelial cell differentiation
neuroepithelial cell differentiation
Count
1.0
1.5
2.0
2.5
3.0
−log10(P-value)
4.5
4.0
3.5
3.0
2.5
2.0
0.025
0.050
0.075
0.100

Figure S2. Reads and RNA information of RNA-seq.

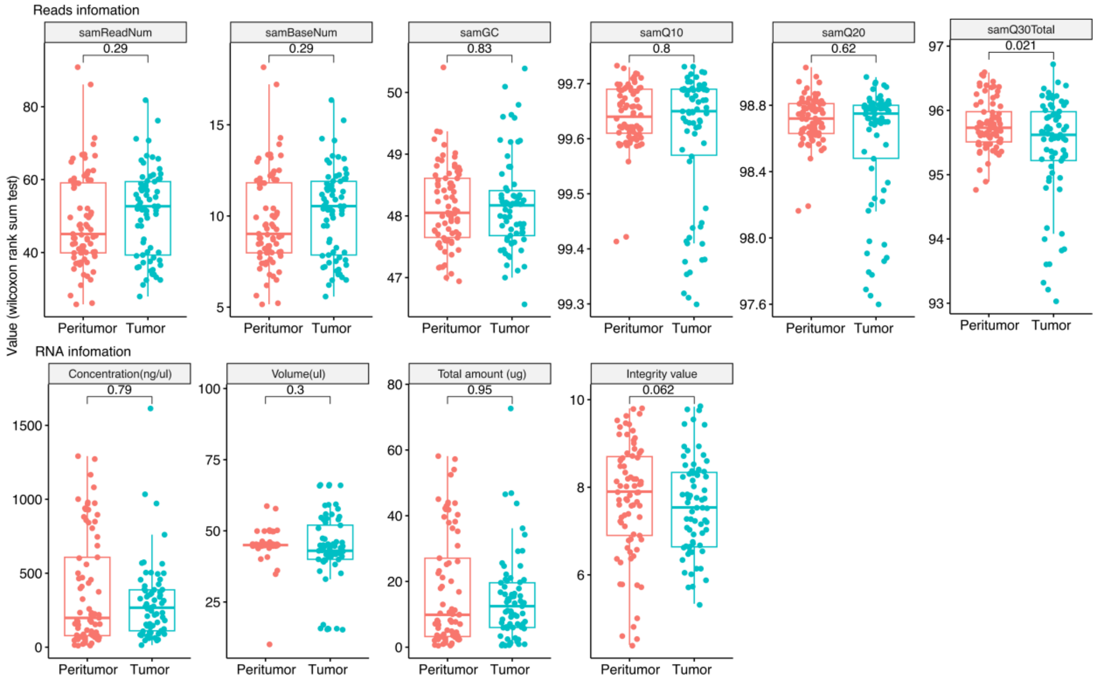

Supplement: Supplementary file 1 [file DataSheet1.pdf]
